# Chuanxiong Formulae for Migraine: A Systematic Review and Meta-Analysis of High-Quality Randomized Controlled Trials

**DOI:** 10.3389/fphar.2018.00589

**Published:** 2018-06-27

**Authors:** Chun-Shuo Shan, Qing-Qing Xu, Yi-Hua Shi, Yong Wang, Zhang-Xin He, Guo-Qing Zheng

**Affiliations:** Department of Neurology, The Second Affiliated Hospital and Yuying Children's Hospital of Wenzhou Medical University, Wenzhou, China

**Keywords:** headache, pain, *Ligusticum chuanxiong* Hort. Root, Traditional Chinese medicine, Chinese herbal medicine

## Abstract

**Objective:** Migraine is a complex, prevalent and disabling neurological disorder characterized by recurrent episodes of headache without ideal treatment. We aim to assess the current available evidence of herbal Chuanxiong (Ligusticum chuanxiong Hort. root) formulae for the treatment of migraine according to the high-quality randomized controlled trials (RCTs).

**Methods:** English and Chinese electronic databases were searched from their inceptions until March 2017. The methodological quality of included study was assessed by the Cochrane Collaboration risk of bias tool. RCTs with Cochrane risk of bias (RoB) score ≥4 were included in the analyses. Meta-analysis was conducted using RevMan 5.3 software. Publication bias was assessed by funnel plot analysis and Egger's test.

**Results:** Nineteen RCTs with 1832 participants were identified. The studies investigated the Chuanxiong formulae vs. placebo (*n* = 5), Chuanxiong formulae vs. conventional pharmacotherapy (CP) (*n* = 13 with 15 comparisons), and Chuanxiong formulae plus CP vs. CP (*n* = 1). Meta-analysis indicated that Chuanxiong formulae could reduce frequency, duration, days and pain severity of migraine and improve the total clinical efficacy rate (*P* < 0.05). Adverse event monitoring was reported in 16 out of 19 studies and occurrence rate of adverse event was low.

**Conclusion:** The findings of present study indicated that Chuanxiong formulae exerted the symptom reliefs of for migraine.

## Introduction

Migraine is characterized as the recurrent episodes of headaches and related symptoms, occurring in 14.70% proportion of population worldwide (Vos et al., 2012). The Global Burden of Disease (GBD) Survey listed migraine as the third most prevalent disorder in 2010 (Vos et al., 2012) and seventh position among the leading causes of disability on a global basis in 2015 (GBD 2015 Disease and Injury Incidence and Prevalence Collaborators, 2016). According to a population-based door-to-door survey of primary headaches in China, the estimated 1-year prevalence of migraine was 9.3% (Yu et al., 2012). The disorder represents a huge socioeconomic burden with a population of over 1.3 billion in China. The total estimated annual cost of primary headache disorders was CNY 672.7 billion, accounting for 2.24% of gross domestic product (GDP) (Yu et al., 2012). Therapeutic agents, including non-steroidal anti-inflammatory drugs (NSAIDs) (aspirin, diclofenac, ibuprofen, naproxen), opioids (butorphanol nasal spray) and triptans (almotriptan; eletriptan; frovatriptan; naratriptan; rizatriptan) are common used in clinic (Carville et al., 2012). In particular, triptans are the first-line acute treatments (Worthington et al., 2013). However, triptans are contraindicated in patients with a history of symptomatic peripheral, coronary, and cerebrovascular disease and severe hypertension (Dodick, 2018). NSAIDs may induce gastrointestinal (Kirthi et al., 2013) and cardiovascular disorders (Moore et al., 2014). Opioids are associated with the incidence of habituation, addiction, tolerance and withdrawal syndromes (Levin, 2014), Furthermore, frequent use of these medications may be contributed to medication-overuse headache (MOH) (Scher et al., 2017). In a word, their applications are still greatly limited by their tolerability and adverse effects. The effective management of headache disorders remains a moving field and a potential challenge to the neurologist (Sinclair et al., 2015). Thus, many migraine patients resort to complementary and alternative medicine (CAM).

Traditional Chinese medicine (TCM), a main form of CAM, has been used for medical treatment of headache in China for the thousands of years and now is still used worldwide. The rhizome of Ligusticum chuanxiong Hort. (Chuanxiong) originated from Divine Husbandman's Classic of the Materia Medica (*Shen Nong Ben Cao Jing*), is a well-known TCM herb (China Pharmacopoeia Committee, 2005). Based on the literature review, Chuanxiong formulae are the most common used Chinese classical and/or patent prescription for treating headache both in ancient and modern time (Zheng Q. et al., 2013; Li et al., 2015). In spite of thousands of years' application history, the efficacy and safety evaluation of Chuanxiong formulae also should be scientifically performed. Previous systematic reviews (Zhou et al., 2013; Li et al., 2015) of TCM for migraine prevented to make firm conclusions because of poor methodological quality of the primary studies. Therefore, the aim of this study is to assess the available evidence of Chuanxiong formulae for migraine according to high-quality randomized controlled trials (RCTs).

## Methods

This systematic review and meta-analysis is reported according to the Preferred Reporting Items for Systematic Reviews and Meta-Analyses: The PRISMA Statement (Moher et al., 2010) and our previous study (Yang et al., 2017).

### Search strategy

PubMed, Cochrane Library, China National Knowledge Infrastructure (CNKI), Chinese Science and Technology Periodical Database (VIP) and Wanfang Database were retrieved in English or in Chinese by using the following search terms: “(migraine OR headache) AND (traditional Chinese medicine OR herbal medicine OR TCM OR integrative medicine OR Integrated Traditional and Western Medicine).” The search time ranged from the inception of each database until March 2017. Moreover, we also manually searched the additional relevant studies, using the references of the systematic reviews that published previously. Specific herb name “Chuanxiong” was not specifically searched to ensure that eligible herbal formulae were included as much as possible.

### Eligibility criteria

Type of participants: The adult participants with migraine of any gender or ethnicity were eligible for inclusion. The widely used diagnosis criteria of headache were Classification and Diagnostic criteria for headache disorders, cranial neuralgias and facial pain (ICHD-1) (Headache Classification Committee of the International Headache Society (IHS), 1988), The international classification of headache disorder, 2nd edition (ICHD-2) (Headache Classification Committee of the International Headache Society (IHS), 2004) and The international classification of headache disorder, 3rd edition (ICHD-3) (Headache Classification Committee of the International Headache Society (IHS), 2013).

Type of study: Only RCTs evaluating the efficacy and safety of Chuanxiong formulae for migraine were eligible. Trials that only mentioned the word “randomization” without any description of the random allocation process were excluded. Quasi-RCTs studies, which allocated participants according to the date of birth, hospital record number, date of admission or identity (ID) number, were also excluded.

Type of intervention: Herbal formulae that must include the herb Chuanxiong was used in the experiment group. There was no limitation on the form of the drug (e.g., liquid, direction, pill, and capsule), dosage, frequency or duration of the treatment. The intervention of control groups included placebo or conventional pharmacotherapy (CP).

Type of outcome measures: The primary outcomes were evaluated by headache frequency, headache duration, headache days and pain intensity. The secondary outcomes measurements were the total clinical effective rate and adverse events.

### Exclusion criteria

Studies were excluded if they did not meet the above eligibility criteria. Additionally, trials with any one of the following conditions were excluded: (1) case series, reviews, observation study, animal researches and pharmacological experiments; (2) duplicated publications; (3) TCM that were used in both treatment group and control group. (4) combined with other CAM therapy, e.g., yoga, massage, Tai Chi, Qigong, acupuncture and moxibustion.

### Study selection

Two reviewers independently screened the titles and abstracts to select eligible RCTs. Full text of the studies that potentially met the predefined criteria were obtained and read. When datasets overlapped or were duplicated, only the most recent information was included. Disagreements about the study selection were resolved by discussing with the corresponding author.

### Data extraction

Two reviewers independently extracted data from the eligible trials using a pre-designed standard data extract form. The following details were extracted: (1) publication year and the first authors' names, publication language, type of headache disorders,diagnosis standard; (2) the characteristics of participants, including number, sex, mean age, course of disease; (3) treatment information, including details of interventions management, course of treatment, follow-up period. (4) outcome measurement and adverse effect. In studies with multiple comparison groups, the most relevant comparison group was chosen for analysis. If outcomes were presented from the studies at different time points, we extracted data from the last time point of treatment. When there were inconsistencies, the corresponding author participated in the extraction. And the original authors of trials were contacted for missing data and additional information.

### Quality assessment

Methodological quality of included studies was assessed by using the risk of bias (RoB) tools in accordance with Cochrane Handbook for Systematic Reviews of Interventions (Higgins et al., 2011). Seven components were as follows: A. adequate sequence generation; B. concealment of allocation; C. blinding (participants and personnel); D. blinding (outcome assessor); E. incomplete outcome data addressed (ITT analysis); F. selective reporting; G. other potential threat to validity. Each of these indicators was categorized as low risk of bias, high risk of bias and unclear. In the scale of zero to seven, we included the studies to enter the final analysis only when they met at least four items. Disagreements between two reviewers about the assessment of quality of included literatures were solved through consultation with corresponding authors.

### Chuanxiong formulae composition

The constituent of Chuanxiong formulae in each included study was recorded. The frequency of use for specific herb was calculated and those with cumulative frequencies over 50% are described in detail.

### Data analysis

Information from eligible studies was aggregated to produce a quantitative summary using the software Cochrane Collaboration Review Manage (RevMan 5.3). Continuous data (headache frequency, headache duration, headache days, pain intensity scales) were expressed as mean difference (MD) or standardized mean difference (SMD) whereas dichotomous data (clinical effective rate) were reported as relative risk (RR) with 95% confidence intervals (CI). Statistical heterogeneity among trials was assessed using the chi-squared test and I^2^ statistic. If no heterogeneity exists (*P* > 0.1, I^2^ < 50%), a fixed effect model (FEM) was applied; otherwise the random effect model (REM) was generally a more plausible match. Sensitivity analysis was performed by changing analysis combination to explore the impact of confounding factors. Meanwhile, in consideration of the differences in participants, interventions and treatment, the subgroup analysis was planned to conduct using the *Z*-test. The differences between the treatment groups and control groups were considered to be statistically significant when *P* < 0.05. If more than10 studies were included in each outcome, funnel plots and Egger's test were used to examine publication bias.

## Results

### Description of studies

**Figure 1:**
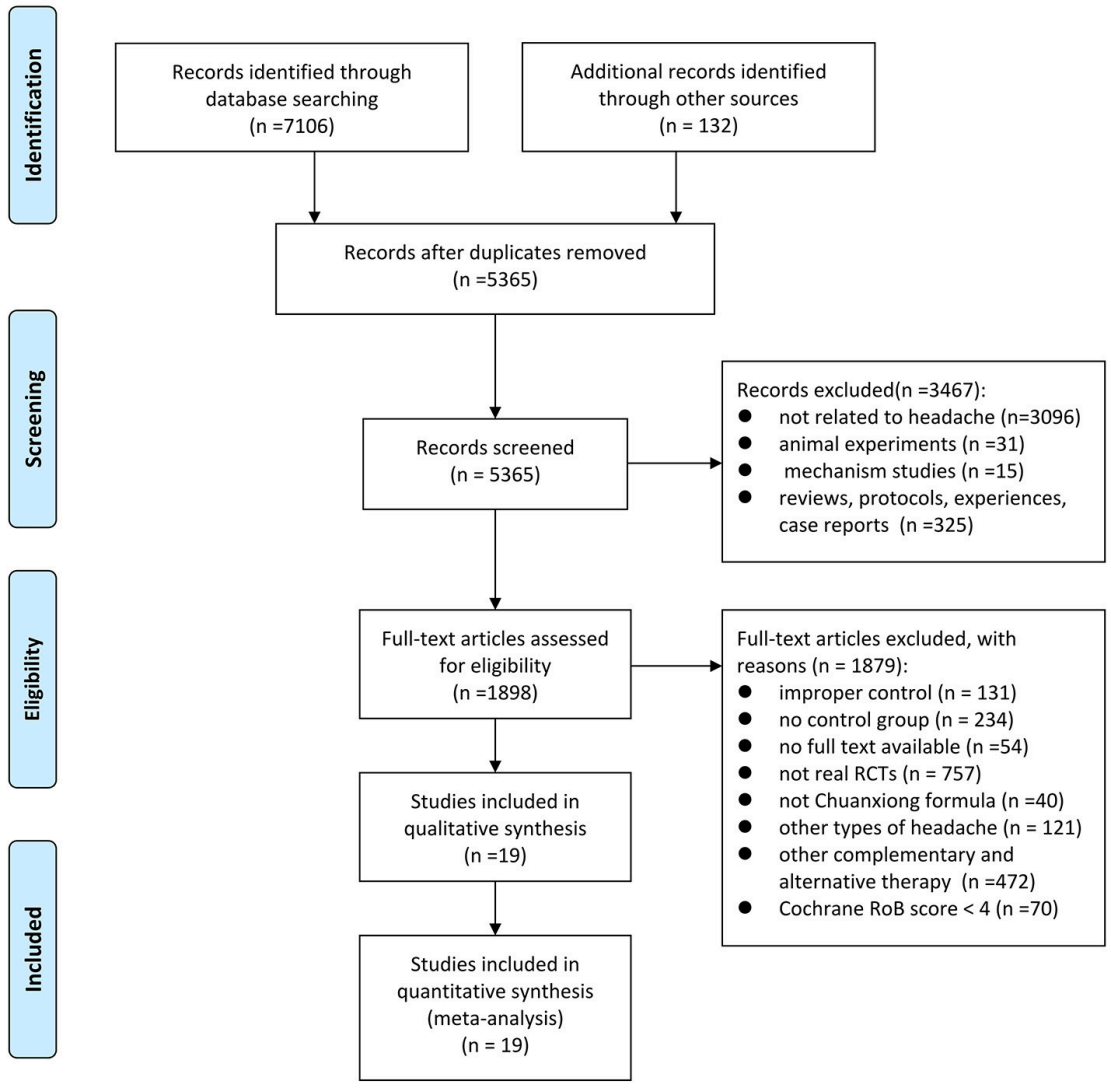
Flow diagram of the search process.

A total of 7238 studies were retrieved through searching five electronic databases and other sources. After duplication removed, 5365 records remained. By screening the titles and abstracts, 3467 records were excluded; among which 3096 studies were not related to headache, 31 papers were animal experiments, 15 were mechanism studies and 325 were reviews, protocols, experiences, or case reports. By reading the full text, 1879 studies were removed, including 131 that had improper control interventions, 234 that were lack of control group, 54 that have no full text available, 757 that were not real RCTs, 40 that did not use Chuanxiong formulae, 121 that were other types of headaches, 472 that contained other CAM therapy, such as acupuncture, massage or scraping, and 70 that had low methodological quality. Ultimately, 19 eligible studies with Cochrane RoB score ≥4 were included for this study (Deng et al., 2001; Luo et al., 2001; Hu et al., 2002; Tan, 2007; Xu, 2011; Fu et al., 2012; Zhang, 2012, 2015; Quan et al., 2013; She, 2013; Cao et al., 2014; Yang, 2014; Guo, 2015; Liang, 2015; Seng, 2015; He and Zhang, 2016; Liu, 2016; Wang et al., 2017; Zhang and Xu, 2017). A PRISMA flow chart depicted the search process and study selection (Figure 1).

### Study characteristics

The characteristics of the 19 included trials with 21 comparisons were summarized in Table 1. All eligible studies were conducted in China. Two articles published in English (Fu et al., 2012; Cao et al., 2014), while the rest of articles published in Chinese (Deng et al., 2001; Luo et al., 2001; Hu et al., 2002; Tan, 2007; Xu, 2011; Zhang, 2012, 2015; Quan et al., 2013; She, 2013; Yang, 2014; Guo, 2015; Liang, 2015; Seng, 2015; He and Zhang, 2016; Liu, 2016; Wang et al., 2017). There were 17 RCTs with two arms (Deng et al., 2001; Luo et al., 2001; Tan, 2007; Xu, 2011; Fu et al., 2012; Zhang, 2012, 2015; She, 2013; Cao et al., 2014; Yang, 2014; Guo, 2015; Liang, 2015; Seng, 2015; He and Zhang, 2016; Liu, 2016; Wang et al., 2017; Zhang and Xu, 2017), 2 RCTs with three arms (Hu et al., 2002; Quan et al., 2013). Two main diagnostic criteria for migraine were ICHD-I and ICHD-II.The sample size of the included studies ranged from 48 to 223, enrolling a total of 1832 participants, 974 patients in treatment groups and 858 patients serving as controls. Five studies compared Chuanxiong formulae alone with placebo (Luo et al., 2001; Xu, 2011; Fu et al., 2012; Cao et al., 2014; Yang, 2014) and 12 studies compared Chuanxiong formulae with CP (Deng et al., 2001; Hu et al., 2002; Tan, 2007; Zhang, 2012, 2015; Quan et al., 2013; She, 2013; Guo, 2015; Liang, 2015; He and Zhang, 2016; Liu, 2016; Wang et al., 2017). Two studies combined Chuanxiong formulae with CP vs. CP (Seng, 2015; Zhang and Xu, 2017). The CP all was Flunarizine Hydrochloride. The preparations used in 19 RCTs with 21 comparisons were administered orally in decoctions (9 comparisons), granules (7 comparisons), capsules (2 comparisons) and pills (3 comparisons). The treatment duration ranged from 1 to 16 weeks. Eleven studies mentioned the duration of follow-up, which lasted from 1 week to 6 months (Deng et al., 2001; Hu et al., 2002; Fu et al., 2012; Zhang, 2012; She, 2013; Cao et al., 2014; Guo, 2015; Liang, 2015; Seng, 2015; Liu, 2016; Wang et al., 2017).

**Table 1:** Basic characteristics of the included studies.

**Included trials**	**Publication language/Headache classification**	**Study design**	**Eligibility criteria**	**No. of participants (male/female); mean age (years)**		**Course of disease**		**Interventions**		**Course of treatment**	**Follow up**	**Outcome index**	**Intergroup differences**
				**Trial**	**Control**	**Trial**	**Control**	**Trial**	**Control**				
Cao et al., 2014	English/Migraine	RCT, Multic-enter	ICHD-II	109 (30/79)38.57 ± 11.93	110 (21/89)38.60 ± 11.56	NR	NR	Zhengtian pill (6 g, tid)	Placebo (6 g, tid)	12 w	4 w	1. Headache frequency 2. Headache duration 3. Headache days	1. NR 2. NR 3. NR
Fu et al., 2012	English/ Migraine	RCT, Multi-center	ICHD-II	86 (23/63)35.77 ± 11.60	42 (11/31)34.58 ± 9.85	86.26 ± 88.10 m	82.12 ±72.76 m	Chuanxiong Ding Tong herbal formula granule (55 g, bid)	Placebo (55 g, bid)	12 w	4 w	1. Headache frequency 2. Headache duration 3. Headache days 4. Pain intensity	1. *P* < 0.05 2. *P* < 0.05 3. *P* < 0.05 4. *P* < 0.05
Deng et al., 2001	Chinese/Migraine	RCT, Single center	ICHD-I	45 (14/31)37.3 ± 8.8	45(16/29)38.8 ± 9.3	4.62 ± 2.85 y	5.02 ± 2.97 y	Toutongkang granules (15 g, bid)	Flunarizine Hydrochloride capsule (5–10 mg, bid or tid)	15 d	6 m	1. Headache frequency 2. Headache duration 3. Pain intensity 4. Total clinical efficacy rate	1. *P* < 0.05 2. *P* < 0.05 3. *P* < 0.05 4. *P* < 0.05
Guo, 2015	Chinese/Migraine	RCT, Single center	ICHD-II	30 (10/20)42.17 ± 12.17	30 (11/19)38.57 ± 9.69	30.67 ± 30.95 m	30.47 ± 27.81 m	Jiawei sanpian decotion (36 g, bid)	Flunarizine Hydrochloride capsule (10 mg, qn)	1 w	1 m	1. Pain intensity 2. Total clinical efficacy rate	1. *P* < 0.05 2. *P* < 0.05
He and Zhang, 2016	Chinese/Migraine	RCT, Single center	ICHD-II	30 (9/21)34.30 ± 15.34	30 (7/23)35.30 ± 16.49	9.20 ± 8.16 y	7.70 ± 5.85 y	Chuanxiong Chatiao San and Qianghuo Shengshi decoction (150 ml, tid)	Flunarizine Hydrochloride capsule (10 mg, qn)	2 w	NR	1. Headache duration 2. Total clinical efficacy rate	1. *P* < 0.05 2. *P* < 0.05
Hu et al., 2002	Chinese/Migraine	RCT, Single center	ICHD-I	30 (9/21)39.83 ± 19.54	30 (10/20)39.12 ± 20.11	8.43 ± 8.56 y	8.20 ± 8.32 y	Shutianning granule (9 g, tid)	Flunarizine Hydrochloride capsule (5 mg, qd)	28 d	1 w	1. Headache frequency 2. Headache duration 3. Pain intensity 4. Total clinical efficacy rate	1. *P* < 0.05 2. *P* < 0.05 3. *P* < 0.05 4. *P* < 0.05
				30 (12/18) 38.92 ± 20.23	30(10/20) 39.12 ± 20.11	7.84 ± 8.80 y	8.20 ± 8.32 y	Fufang Yangjiao capsule (1.25 mg, tid)	Flunarizine Hydrochloride capsule (5 mg, qd)	28 d	1 w	1. Headache frequency 2. Headache duration 3. Pain intensity 4. Total clinical efficacy rate	1. *P* < 0.05 2. *P* < 0.05 3. *P* < 0.05 4. *P* < 0.05
Liang, 2015	Chinese/Migraine	RCT, Multi-center	ICHD-II	113 (29/84) 35.35 ± 10.87	110 (24/86) 34.01 ± 9.06	77.20 ± 45.09 m	73.95 ± 38.94 m	He Jie Zhi Tong Decoction (100 ml, bid)	Flunarizine Hydrochloride capsule (10 mg, qn)	8 w	4 w	1. Headache frequency 2. Headache duration 3. Headache days 4. Pain intensity 5. Total clinical efficacy rate	1. *P* < 0.05 2. *P* < 0.05 3. *P* < 0.05 4. *P* < 0.05 5. *P* < 0.05
Liu, 2016	Chinese/Migraine	RCT, Single center	ICHD-II	30 (7/23) 42.9 ± 11.74	30 (10/20) 46.9 ± 12.29	75.82 ± 33.61 m	74.95 ± 38.18 m	Toutongning pill (6 g, tid)	Flunarizine Hydrochloride capsule (10 mg, qn)	16 w	1 m	1. Headache frequency 2. Headache duration 3. Pain intensity 4. Total clinical efficacy rate	1. *P* < 0.05 2. *P* < 0.05 3. *P* < 0.05 4. *P* < 0.05
Luo et al., 2001	Chinese/Migraine	RCT, Multi-center	NR	56 (22/34) 38.5 ± 8.6	56 (20/36) 37.6 ± 11.0 y	NR	NR	Yangxueqingnao granule (4 g,tid)	Flunarizine Hydrochloride capsule (4 g, tid)	30 d	NR	1. Headache frequency 2. Headache duration 3. Total clinical efficacy rate	1. *P* < 0.05 2. *P* < 0.05 3. *P* < 0.05
Quan et al., 2013	Chinese/Migraine	RCT, Single center	ICHD-II	43 (20/23) 34.53 ± 8.86	38 (20/18) 33.55 ± 9.39	11.40 ± 7.44 y	11.24 ± 7.50 y	High-dose Tianning yin (200 ml, bid)	Flunarizine Hydrochloride capsule (5 mg, qn)	30 d	NR	1. Headache frequency 2. Headache duration 3. Pain intensity 4. Total clinical efficacy rate	1. *P* < 0.05 2. *P* < 0.05 3. *P* < 0.05 4. *P* < 0.05
				45 (22/23) 34.38 ± 8.34	38 (20/18) 33.55 ± 9.39	10.31 ± 6.82 y	11.24 ± 7.50 y	Low-dose Tianning yin (200 ml, bid)	Flunarizine Hydrochloride capsule (5 mg, qn)	30 d	NR	1. Headache frequency 2. Headache duration 3. Pain intensity 4. Total clinical efficacy rate	1. *P* < 0.05 2. *P* < 0.05 3. *P* < 0.05 4. *P* < 0.05
Seng, 2015	Chinese/Migraine	RCT, Single center	ICHD-II	30 (8/22) 44.00 ± 8.51	39 (20/18) 43.77 ± 8.86	43.92 ± 17.75 m	41.53 ± 21.06 m	1.Xiaotong decoction (200 mg, bid); 2 Flunarizine Hydrochloride capsule (10 mg, qn)	Flunarizine Hydrochloride capsule (10 mg, qn)	60 d	1 m	1. Total clinical efficacy rate	1. *P* < 0.05
She, 2013	Chinese/Migraine	RCT, Single center	ICHD-II	36 (12/24) 41.25 ± 11.83	36 (10/26) 40.01 ± 12.02	7.39 ± 4.61 y	7.11 ± 5.39 y	Toutongning mixture (100 ml, bid)	Flunarizine Hydrochloride capsule (5 mg, qn)	14 d	4 w	1. Headache frequency 2. Headache duration 3. Headache days 4. Pain intensity 5. Total clinical efficacy rate	1. *P* < 0.05 2. *P* < 0.05 3. *P* > 0.05 4. *P* > 0.05 5. *P* > 0.05
Tan, 2007	Chinese/Migraine	RCT, Single center	ICHD-II	40 (13/27) 38.13 ± 3.65	40 (15/25) 37.86 ± 4.28	6.17 ± 1.79 y	5.91 ± 2.62 y	Tongqiao Zhitong pill (5 g, bid)	Flunarizine Hydrochloride capsule (10 mg, qn)	4 w	NR	1. Total clinical efficacy rate	1. *P* < 0.05
Wang et al., 2017	Chinese/Migraine	RCT, Single center	CCEDTM	30(7/23) 46.3 ± 13.3	30 (8/22) 48.3 ± 13.07	1–11 y	1–12 y	Pinggan Huoxue decoction granule (1/2 dose, bid)	Flunarizine Hydrochloride capsule (5 mg, qn)	14 d	1 m	1. Pain intensity 2. Total clinical efficacy rate	*1. P* < 0.05 2. *P* < 0.05
Xu, 2011	Chinese/Migraine	RCT, Single center	ICHD-II	24(5/19) NR	24 (11/13) NR	NR	NR	Migraine granule (1/2 dose, bid)	Placebo (1/2 dose, bid)	12 w	NR	1. Headache frequency 2. Headache duration 3. Pain intensity 4. Total clinical efficacy rate	1. *P* < 0.05 2. *P* < 0.05 3. *P* < 0.05 4. *P* < 0.05
Yang, 2014	Chinese/Migraine	RCT, Single center	ICHD-II	30 (7/23) 41.581 ± 12.50	30 (10/20) 40.229 ± 13.73	75.82 ± 33.61 m	74.95 ± 38.18 m	Wind-dispelling and Pain-relieving capsule (4 capsule, tid)	Placebo (4 capsule, tid)	12 w	NR	1. Headache frequency 2. Headache duration 3. Pain intensity 4. Total clinical efficacy rate	1. *P* < 0.05 2. *P* < 0.05 3. *P* < 0.05 4. *P* < 0.05
Zhang and Xu, 2017	Chinese/Migraine	RCT, Single center	NR	44 (19/25) 39.11 ± 7.28	44 (20/24) 38.65 ± 7.41	8.35 ± 5.46 y	8.41 ± 5.33 y	1. Xiongchong sanpian decoction(200 ml, bid) 2. Flunarizine Hydrochloride capsule (10 mg, qn)	Flunarizine Hydrochloride capsule (10 mg, qn)	3 m	NR	1. Headache frequency 2. Headache duration 3. Pain intensity 4. Total clinical efficacy rate	1. *P* < 0.05 2. *P* < 0.05 3. *P* < 0.05 4. *P* < 0.05
Zhang, 2012	Chinese/Migraine	RCT, Multi-center	ICHD-II	60 (24/36) 38.00 ± 11.33	60 (16/44) 37.03 ± 11.64	24.55 ± 19.25 m	29.37 ± 22.57 y	Xiongzhi Zhentong granules (1/2 dose, bid)	Flunarizine Hydrochloride capsule (5 mg, qn)	14 d	1 m	1. Pain intensity 2. Total clinical efficacy rate	1. *P* < 0.05 2. *P* < 0.05
Zhang, 2015	Chinese/Migraine	RCT, Single center	ICHD-II	33 (13/20) NR	34 (15/19) NR	NR	NR	Shugan Tongluo II Prescription (150 ml, bid)	Flunarizine Hydrochloride capsule (5 mg, qn)	30 d	NR	1. Headache frequency 2. Pain intensity	1. *P* < 0.05 2. *P* > 0.05

*bid, bis in die; CCEDTM, Chinese consensus of experts on diagnosis and treatment of migraine; d, day; g, gram; ICHD-I, Classification and Diagnostic criteria for headache disorders, cranial neuralgias and facial pain; ICHD-II, The international classification of headache disorder, 2nd edition; m, month; mg, milligram; ml, milliliter; NR, not reported; qd, quaque die; qn, quaque nocte; RCT, Randomized Controlled Trial; tid, ter in die; w, week; y, year*.

### Description of the Chuanxiong formulae

The constituent of Chuanxiong formulae in each included study was detailed in Table 2. Sixty-four herbs were used in the 19 different Chuanxiong formulae. The top 12 most frequently used herbs were ordinally Rhizoma Ligustici Chuanxiong (sichuan lovage rhizome), Radix Angelicae Dahuricae (dahurian angelica root), Ramulus Uncariae Cum Uncis (gambir plant nod), Herba Asari (manchurian wildginger), Radix Angelicae Sinensis (Chinese angelica), Scorpio (scorpion), Radix Glycyrrhizae (liquorice root), Radix Paeoniae Alba (debark peony root), Flos Carthami (safflower), Radix Cyathulae (medicinal cyathula root), Radix Paeoniae Rubra (peony root), Rhizoma Corydalis (yanhusuo), which were used more than 4 times (Table 3).

**Table 2:** The constituent of Chuanxiong formulae in the included studies.

**Included trials**	**Chuanxiong formula**	**Ingredients**		
		**Latin name**	**English name**	**Chinese name**
Cao et al., 2014	Zhengtian pill	Rhizoma Ligustici Chuanxiong	Sichuan lovage rhizome	Chuanxiong
		Rhizoma et Radix Notopterygii	Incised notopterygium rhizome and root	Qianghuo
		Radix Saposhnikoviae	Divaricate saposhnikovia root	Fangfeng
		Radix Angelicae Dahuricae	Dahurian angelica root	Baizhi
		Ramulus Uncariae Cum Uncis	Gambir plant nod	Gouteng
		Semen Persicae	Peach seed	Taoren
		Flos Carthami	Safflower	Honghua
		Radix Angelicae Sinensis	Chinese angelica	Danggui
		Caulis Spatholobi	Suberect spatholobus stem	Jixueteng
		Radix Rehmanniae Recens	Unprocessed rehmannia root	Dihuang
		Radix Angelicae Pubescentis	Doubleteeth pubescent angelica root	Duhuo
		Radix Aconiti Lateralis Preparata	Prepared common monkshood branched	Fupian
		Herba Ephedrae	Root ephedra	Mahuang
		Herba Asari	Manchurian wildginger	Xixin
		Radix Paeoniae Alba	Debark peony root	Baishao
Fu et al., 2012	Chuanxiong Ding Tong herbal formula granule	Rhizoma Ligustici Chuanxiong	Sichuan lovage rhizome	Chuanxiong
		Radix Cyathulae	Medicinal cyathula root	Chuanniuxi
		Rhizoma Dioscoreae Hypoglaucae	Poison yam	Chuanbixie
		Flos Chrysanthemi	Chrysanthemum flower	Juhua
		Ramulus Uncariae Cum Uncis	Gambir plant nod	Gouteng
		Fructus Tribuli	Puncturevine caltrop fruit	Baijili
		Semen Coicis	Coix seed	Yiyiren
		Fructus Ammomi Rotundus	Cardamon fruit	Baidoukou
		Rhizoma Pinelliae Preparatum	Processed pinellia tuber	Zhibanxia
Deng et al., 2001	Toutongkang granules	Rhizoma Ligustici Chuanxiong	Sichuan lovage rhizome	Chuanxiong
		Flos Carthami	Safflower	Honghua
		Radix Angelicae Sinensis	Chinese angelica	Danggui
		Radix Salviae Miltiorrhizae	Danshen root	Danshen
		Radix Puerariae	Kudzuvine root	Gegen
		Scorpio	Scorpion	Quanxie
		Rhizoma Acori Tatarinowii	Grassleaf sweetflag rhizome	Shichangpu
		Rhizoma Corydalis	Yanhusuo	Yanhusuo
Guo, 2015	Jiawei sanpian decotion	Rhizoma Ligustici Chuanxiong	Sichuan lovage rhizome	Chuanxiong
		Radix Paeoniae Alba	Debark peony root	Baizhi
		Semen Sinapis Albae	Mustard	Baijiezi
		Rhizoma Cyperi	Nutgrass galingale rhizome	Xiangfu
		Radix Angelicae Dahuricae	Dahurian angelica root	Baishao
		Scorpio	Scorpion	Quanchong
He and Zhang, 2016	Chuanxiong Chatiao San and Qianghuo Shengshi decoction	Rhizoma Ligustici Chuanxiong	Sichuan lovage rhizome	Chuanxiong
		Herba Schizonepetae	Fineleaf schizonepeta herb	Jingjie
		Radix Saposhnikoviae	Divaricate saposhnikovia root	Fangfeng
		Radix Angelicae Dahuricae	Dahurian angelica root	Baizhi
		Herba Asari	Manchurian wildginger	Xixin
		Herba Menthae	Peppermint	Bohe
		Rhizoma et Radix Notopterygii	Incised notopterygium rhizome and root	Qianghuo
		Fructus Viticis	Shrub chastetree fruit	Manjingzi
		Rhizoma Ligustici	Chinese lovage	Gaoben
		Radix Glycyrrhizae	Liquorice root	Gancao
Hu et al., 2002 a	Shutianning granule	Rhizoma Gastrodiae	Tall gastrodia tuber	Tianma
		Herba Selaginellae	Spikemoss	Juanbai
		Fructus Gardeniae	Cape jasmine fruit	Zhizi
		Rhizoma Ligustici Chuanxiong	Sichuan lovage rhizome	Chuanxiong
		Radix Angelicae Dahuricae	Dahurian angelica root	Baizhi
		Fructus Aurantii Immaturus	Immature orange fruit	Zhishi
		Concha Margaritifera	Nacre	Zhenzhumu
Hu et al., 2002 b	Fufang Yangjiao capsule	Cornu Saigae Tataricae	Antelope horn	Yangjiao
		Rhizoma Ligustici Chuanxiong	Sichuan lovage rhizome	Chuanxiong
		Radix Angelicae Dahuricae	Dahurian angelica root	Baizhi
		Radix Polygoni Multiflori Preparata	Prepared fleeceflower root	Zhishouwu
Liang, 2015	He Jie Zhi Tong Decoction	Radix Bupleuri	Chinese thorowax root	Chaihu
		Rhizoma Ligustici Chuanxiong	Sichuan lovage rhizome	Chuanxiong
		Radix Scutellariae	Baical skullcap root	Huangqin
		Rhizoma Pinelliae Preparata	Alum processed pinellia	Qingbanxia
		Radix Codonopsis	Tangshen	Dangshen
		Rhizoma Atractylodis Macrocephalae	Largehead atractylodes rhizome	Baishu
		Radix Glycyrrhizae	Liquorice root	Gancao
		Os Draconis	Bone fossil of big mammals	Longgu
		Radix Polygalae	Milkwort root	Yuanzhi
		Scorpio	Scorpion	Quanxie
		Scolopendra	Centipede	Wugong
Liu, 2016	Toutongning pill	Radix Astragali seu Hedysari	Milkvetch root	Huangqi
		Radix Paeoniae Rubra	Peony root	Chishao
		Rhizoma Ligustici Chuanxiong	Sichuan lovage rhizome	Chuanxiong
		Radix Angelicae Sinensis	Chinese angelica	Danggui
		Herba Asari	Manchurian wildginger	Xixin
Luo et al., 2001	Yangxueqingnao granule	Radix Angelicae Sinensis	Chinese angelica	Danggui
		Rhizoma Ligustici Chuanxiong	Sichuan lovage rhizome	Chuanxiong
		Radix Paeoniae Alba	Debark peony root	Baishao
		Radix Rehmanniae Preparata	Prepared rehmannia root	Shudihuang
		Ramulus Uncariae Cum Uncis	Gambir plant nod	Gouteng
		Caulis Spatholobi	Suberect spatholobus stem	Jixueteng
		Spica Prunellae	Common selfheal fruit-spike	Xiakucao
		Semen Cassiae	Cassia seed	Juemingzi
		Concha Margaritifera	Nacre	Zhenzhumu
		Rhizoma Corydalis	Yanhusuo	Yanhusuo
		Herba Asari	Manchurian wildginger	Xixin
Quan et al., 2013	Tianning yin	Rhizoma Ligustici Chuanxiong	Sichuan lovage rhizome	Chuanxiong
		Radix Angelicae Dahuricae	Dahurian angelica root	Baizhi
		Ramulus Uncariae Cum Uncis	Gambir plant nod	Gouteng
		Radix Paeoniae Rubra	Peony root	Chishao
		Bombyx Batryticatus	Stiff silkworm	Jiangcan
		Scorpio	Scorpion	Zhiquanxie
Seng, 2015	Xiaotong decoction	Rhizoma Ligustici Chuanxiong	Sichuan lovage rhizome	Chuanxiong
		Radix Angelicae Dahuricae	Dahurian angelica root	Baizhi
		Herba Asari	Manchurian wildginger	Xixin
		Semen Sinapis Albae	Mustard seed	Baijiezi
		Scorpio	Scorpion	Quanxie
		Radix Glehniae	Coastal glehnia root	Beishasheng
		Fructus Viticis	Shrub chastetree fruit	Manjingzi
		Herba Schizonepetae	Fineleaf schizonepeta herb	Jingjie
		Rhizoma Smilacis Glabrae	Glabrous greenbrier rhizome	Tufuling
		Radix Glycyrrhizae	Liquorice root	Gancao
She, 2013	Toutongning mixture	Rhizoma Gastrodia	Tall gastrodia tuber	Tianma
		Herba Asari	Manchurian wildginger	Xixin
		Rhizoma Ligustici Chuanxiong	Sichuan lovage rhizome	Chuanxiong
		Ramulus Uncariae Cum Uncis	Gambir plant nod	Gouteng
		Radix Angelicae Dahuricae	Dahurian angelica root	Baizhi
		Radix Angelicae Sinensis	Radix Angelicae Sinensis	Danggui
		Lumbricus	Earthworm	Dilong
		Radix Achyranthis Bidentatae	Twotoothed achyranthes root	Niuxi
Tan, 2007	Tongqiao Zhitong pill	Olibanum	Frankincense	Ruxiang
		Myrrha	Myrrh	Moyao
		Semen Persicae	Peach seed	Taoren
		Flos Carthami	Safflower	Honghua
		Rhizoma Ligustici Chuanxiong	Sichuan lovage rhizome	Chuanxiong
		Radix Bupleuri	Chinese thorowax root	Chaihu
		Radix et Rhizoma Nardostachyos	Nardostachys root	Gansong
		Radix Angelicae Dahuricae	Dahurian angelica root	Baizhi
Wang et al., 2017	Pinggan Huoxue decoction granule	Fructus Tribuli	Puncturevine caltrop fruit	Jili
		Radix Bupleuri	Chinese thorowax root	Chaihu
		Rhizoma Cyperi	Nutgrass galingale rhizome	Xiangfu
		Rhizoma Ligustici Chuanxiongchuan	Sichuan lovage rhizome	Chuanxiong
		Radix Angelicae Dahuricae	Dahurian angelica root	Baizhi
		Rhizoma Corydalis	Yanhusuo	Yanhusuo
		Radix Paeoniae Alba	Debark peony root	Baishao
		Caulis Polygoni Multiflori	Tuber fleeceflower stem	Yejiaoteng
		Concha Ostreae	Oyster shell	Muli
		Radix Puerariae	Kudzuvine root	Gegen
Xu, 2011	Migraine granule	Rhizoma Ligustici Chuanxiongchuan	Sichuan lovage rhizome	Chuanxiong
		Radix Cyathulae	Medicinal cyathula root	Chuanniuxi
		Rhizoma Dioscoreae Hypoglaucae	Poison yam	Chuanbixie
		Flos Chrysanthemi	Chrysanthemum flower	Juhua
		Ramulus Uncariae Cum Uncis	Gambir plant nod	Gouteng
		Fructus Tribuli	Puncturevine caltrop fruit	Jili
		Semen Coicis	Coix seed	Yiyiren
		Fructus Ammomi Rotundus	Cardamon fruit	Baidoukou
		Rhizoma Pinelliae Preparatum	Processed pinellia tuber	Fabanxia
				
Yang, 2014	Wind-dispelling and Pain-relieving capsule	Rhizoma Ligustici Chuanxiong	Sichuan lovage rhizome	Chuanxiong
		Radix Angelicae Dahuricae	Dahurian angelica root	Baizhi
		Fructus Evodiae	Medicinal evodia fruit	Wuzhuyu
		Herba Menthae	Peppermint	Bohenao
Zhang and Xu, 2017	Xiongchong sanpian decoction	Rhizoma Ligustici Chuanxiong	Sichuan lovage rhizome	Chuanxiong
		Scorpio	Scorpion	Quanxie
		Ramulus Uncariae Cum Uncis	Gambir plant nod	Gouteng
		Radix Salviae Miltiorrhizae	Danshen root	Danshen
		Radix Achyranthis Bidentatae	Twotoothed achyranthes root	Niuxi
		Eupolyphaga Seu Steleophaga	Ground beetle	Tubiechong
		Rhizoma Corydalis	Yanhusuo	Yanhusuo
		Radix Angelicae Dahuricae	Dahurian angelica root	Baizhi
		Herba Asari	Manchurian wildginger	Xixin
		Fructus Viticis	Shrub chastetree fruit	Manjinzi
		Radix Glycyrrhizae	Liquorice root	Gancao
Zhang, 2012	Xiongzhi Zhentong granules	Rhizoma Ligustici Chuanxiong	Sichuan lovage rhizome	Chuanxiong
		Radix Angelicae Sinensis	Chinese angelica	Danggui
		Radix Angelicae Dahuricae	Dahurian angelica root	Baizhi
		Bombyx Batryticatus	Stiff silkworm	Jiangcan
		Radix Glycyrrhizae	Liquorice root	Gancao
Zhang, 2015	Shugan Tongluo II prescription	Radix Angelicae Sinensis	Chinese angelica	Danggui
		Radix Paeoniae Alba	Debark peony root	Baishao
		Rhizoma Gastrodiae	Tall gastrodia tuber	Tianma
		Cornu Bubali	Buffalo horn	Shuiniujiao
		Rhizoma Ligustici Chuanxiong	Sichuan lovage rhizome	Chuanxiong
		Radix Angelicae Dahuricae	Dahurian angelica root	Baizhi
		Flos Carthami	Safflower	Honghua
		Herba Asari	Manchurian wildginger	Xixin

**Table 3:** Analysis of the top 12 frequency Chinese herb medicine in treatment of migraine.

**Herb name Latin (English)**	**Frequency**	**The total frequency (%)**	**Cumulative percentiles (%)**
Rhizoma Ligustici Chuanxiong (sichuan lovage rhizome)	21	12.14	12.14
Radix Angelicae Dahuricae (dahurian angelica root)	16	9.25	21.39
Ramulus Uncariae Cum Uncis (gambir plant nod)	9	5.20	26.59
Herba Asari (manchurian wildginger)	8	4.62	31.21
Radix Angelicae Sinensis (Chinese angelica)	7	4.05	35.26
Scorpio (scorpion)	6	3.47	38.73
Radix Glycyrrhizae (liquorice root)	5	2.89	41.62
Radix Paeoniae Alba (debark peony root)	5	2.89	44.51
Flos Carthami(safflower)	4	2.31	46.82
Radix Cyathulae (medicinal cyathula root)	4	2.31	49.13
Radix Paeoniae Rubra (peony root)	4	2.31	51.45
Rhizoma Corydalis (yanhusuo)	4	2.31	53.76

### RoB

RoB assessment is shown in Table 4. All included studies were described as “randomized” with appropriate methods of sequence generation. Twelve studies used a random number table in the allocation of participants (Deng et al., 2001; Luo et al., 2001; Hu et al., 2002; Tan, 2007; Quan et al., 2013; She, 2013; Guo, 2015; Seng, 2015; Zhang, 2015; Liu, 2016; Wang et al., 2017; Zhang and Xu, 2017); three studies applied Statistical Analysis System (SAS) software (Zhang, 2012; Liang, 2015; He and Zhang, 2016); two studies were central assignment (Xu, 2011; Fu et al., 2012); one study employed Statistical Product and Service Solutions (SPSS) software to generate the random numbers (Yang, 2014) and another one mentioned randomization by computer-generated stochastic system (Cao et al., 2014). These 19 studies were assessed to be low RoB in the domain of sequence generation. One study applied “sealed envelopes” (He and Zhang, 2016) and two studies applied central allocation concealment in the trial design (Xu, 2011; Fu et al., 2012). Five studies were double blindness (Luo et al., 2001; Xu, 2011; Fu et al., 2012; Cao et al., 2014; Yang, 2014). All studies either had dropouts with adequate explanations and appropriate methods to treat missing data or had no dropouts. Finally, 16 out of 19 studies were at low RoB from other sources including funding, protocols, conflicts of interest, and baseline balance (Deng et al., 2001; Hu et al., 2002; Tan, 2007; Xu, 2011; Fu et al., 2012; Zhang, 2012, 2015; Quan et al., 2013; She, 2013; Yang, 2014; Guo, 2015; Liang, 2015; Seng, 2015; Liu, 2016; Wang et al., 2017; Zhang and Xu, 2017), except for 3 studies that did not reported available funding or protocols was therefore at unclear RoB (Luo et al., 2001; Cao et al., 2014; He and Zhang, 2016).

**Table 4:** Risk of bias assessments for included studies.

**Included studies**	**A**	**B**	**C**	**D**	**E**	**F**	**G**	**Total**
Cao et al., 2014	+	?	+	?	+	?	+	4
Deng et al., 2001	+	?	–	?	+	+	+	4
Fu et al., 2012	+	+	+	?	+	+	+	6
Guo, 2015	+	?	–	?	+	+	+	4
He and Zhang, 2016	+	+	–	?	+	?	+	4
Hu et al., 2002	+	?	–	+	+	+	+	5
Liang, 2015	+	?	–	?	+	+	+	4
Liu, 2016	+	?	–	?	+	+	+	4
Luo et al., 2001	+	?	+	?	+	?	+	4
Quan et al., 2013	+	?	–	?	+	+	+	4
Seng, 2015	+	?	–	?	+	+	+	4
She, 2013	+	?	–	?	+	+	+	4
Tan, 2007	+	?	–	?	+	+	+	4
Wang et al., 2017	+	–	–	–	+	+	+	4
Xu, 2011	+	+	+	?	+	+	+	6
Yang, 2014	+	?	+	?	+	–	+	4
Zhang and Xu, 2017	+	–	–	–	+	+	+	4
Zhang, 2012	+	?	–	?	+	+	+	4
Zhang, 2015	+	?	–	?	+	+	+	4

*A, adequate sequence generation; B, concealment of allocation; C, Blinding of participants and personnel; D, Blinding of out-come assessment; E, Incomplete out-come data; F, Selective reporting; G, Other bias; +, low risk of bias, –, high risk of bias, ?, unclear risk of bias*.

### Effectiveness

#### Migraine frequency

**Figure 2:**
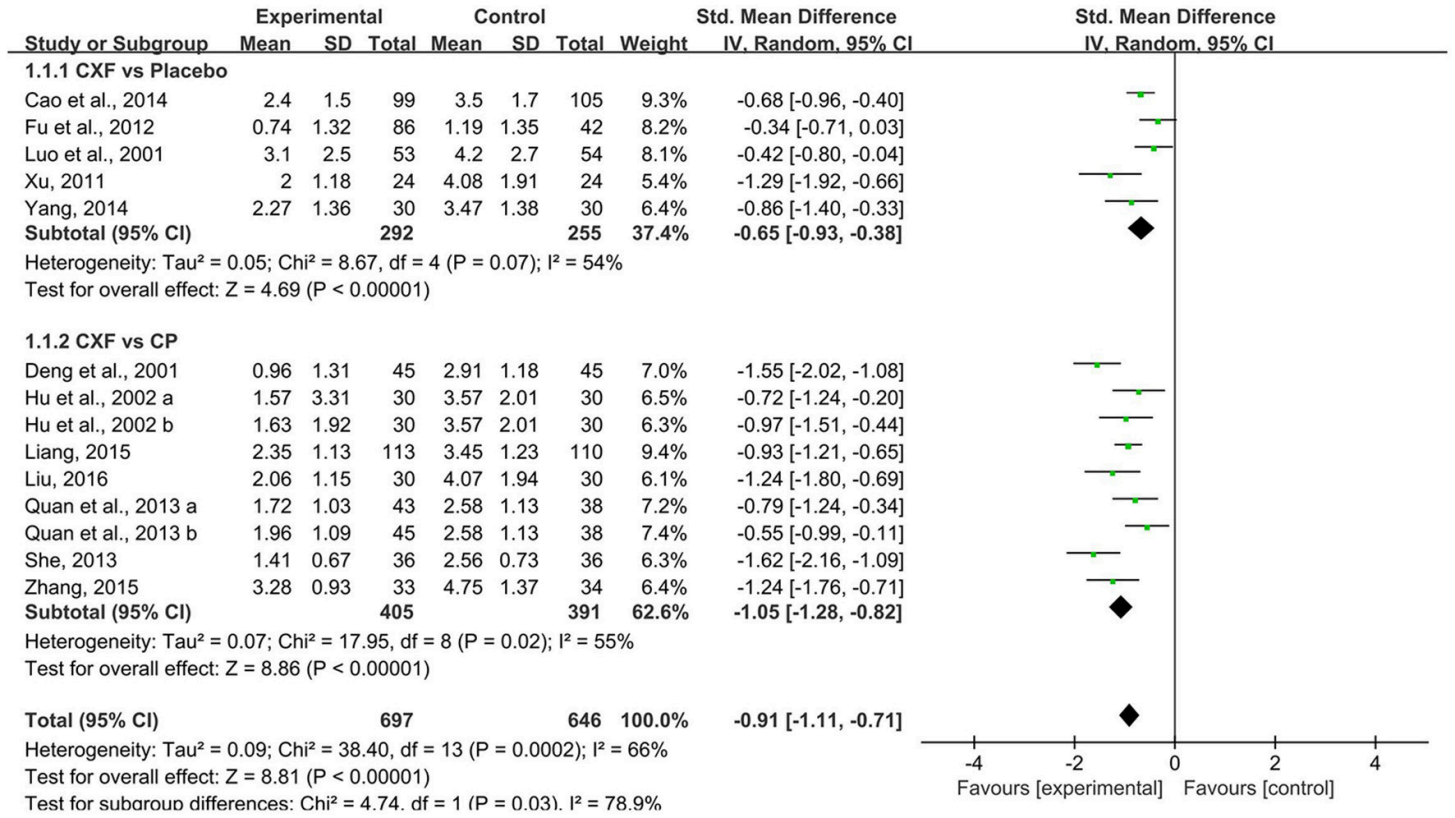
The forest plot of the efficacy of Chuanxiong formulae on the migraine frequency. CXF, Chuanxiong formulae; CP, conventional pharmacotherapy.

Thirteen studies evaluated the frequency of migraine attack in a month, and data showed a significant reduction both in studies that compared with placebo (SMD = −0.65, 95% CI −0.93 to −0.38, *P* < 0.00001, heterogeneity χ^2^ = 8.67, *P* = 0.07, I^2^ = 54%, Figure 2; Luo et al., 2001; Xu, 2011; Fu et al., 2012; Cao et al., 2014; Yang, 2014) and compared with CP (SMD = −1.05, 95% CI −1.28 to −0.82, *P* < 0.00001, heterogeneity χ^2^ = 17.95, *P* = 0.02, I^2^ = 55%, Figure 2; Deng et al., 2001; Hu et al., 2002; Quan et al., 2013; She, 2013; Liang, 2015; Zhang, 2015; Liu, 2016). Only one study (Zhang and Xu, 2017) compared Chuanxiong formulae plus CP with CP alone. The result of the study favored the combined treatment with *P* < 0.05.

#### Migraine duration

**Figure 3:**
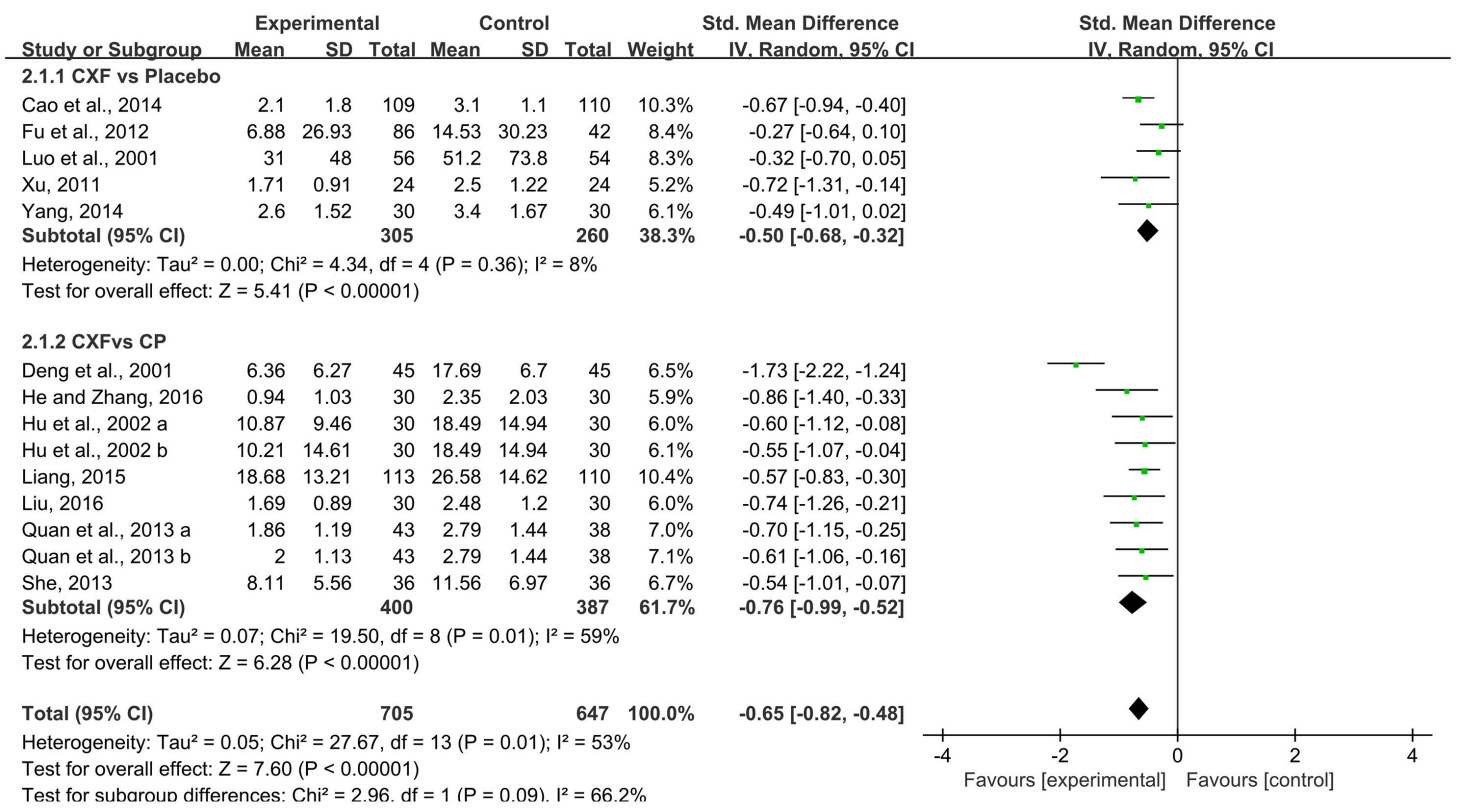
The forest plot of the efficacy of Chuanxiong formulae on the migraine duration. CXF, Chuanxiong formulae; CP, conventional pharmacotherapy.

There were 12 trials with 14 comparisons reported headache duration as outcome measure. Meta-analysis demonstrated that Chuanxiong formulae were significantly better at reducing the duration of migraine than placebo (SMD = −0.50, 95% CI −0.68 to −0.32, *P* < 0.00001, heterogeneity χ^2^ = 4.34, *P* = 0.36, *I*^2^ = 8%, Figure 3; Xu, 2011; Fu et al., 2012; Cao et al., 2014; Yang, 2014) and CP (SMD = −0.76, 95% CI −0.99 to −0.52, *P* < 0.00001, heterogeneity χ^2^ = 19.50, *P* = 0.01, *I*^2^ = 59%, Figure 3; Deng et al., 2001; Hu et al., 2002; Quan et al., 2013; She, 2013; Liang, 2015; He and Zhang, 2016; Liu, 2016). There was homogeneity for this outcome in the placebo comparison but not in the Chuanxiong formulae vs. CP comparison. After excluding one study (Deng et al., 2001) which had relatively short course of disease, the result still indicated a benefit in the Chuanxiong formulae groups (SMD −0.62, 95% CI −0.78 to −0.47, *P* < 0.00001, heterogeneity χ^2^ = 1.47, *P* = 0.98, *I*^2^ = 0%). For the comparison of Chuanxiong formulae plus CP vs. CP, one study (Zhang and Xu, 2017) demonstrated that combined treatment had better effect than conventional medicine alone (*P* < 0.05).

#### Migraine days

**Figure 4:**
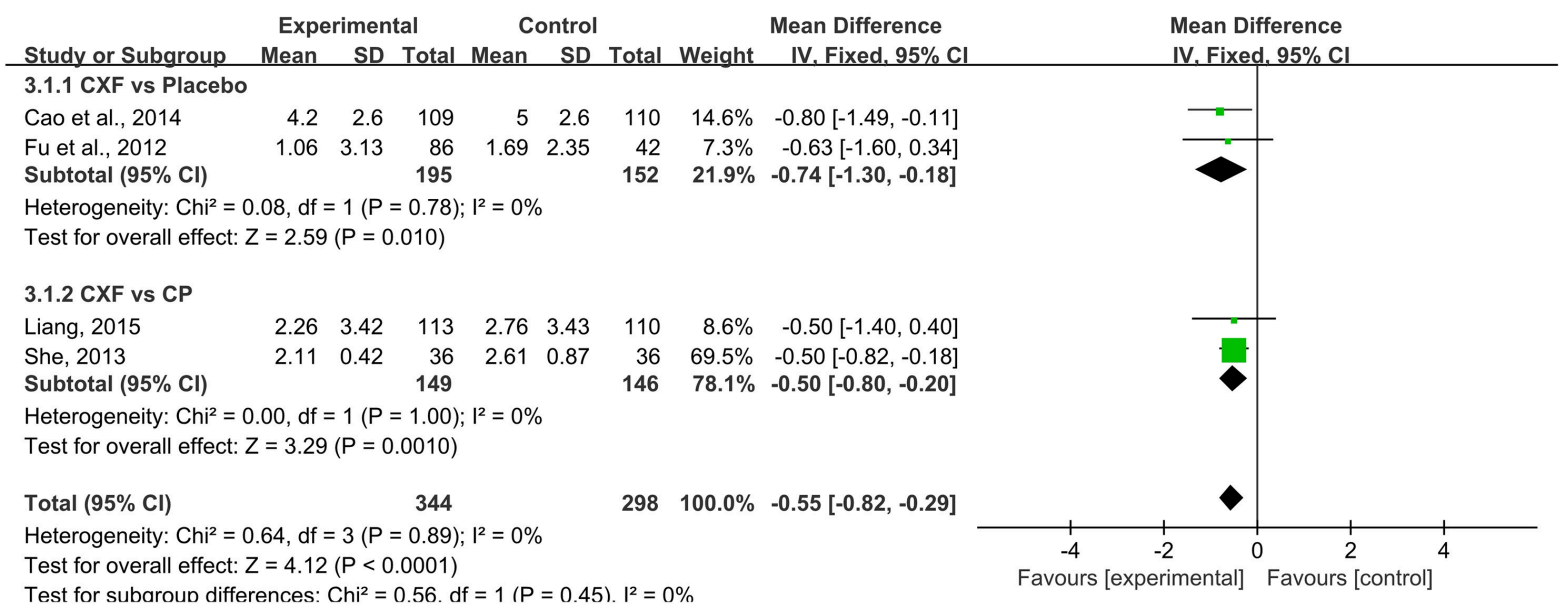
The forest plot of the efficacy of Chuanxiong formulae on the migraine days. CXF, Chuanxiong formulae; CP, conventional pharmacotherapy.

Four studies analyzed showed a statistically significant difference in the outcome of migraine days. For two multi-center RCTs (Fu et al., 2012; Cao et al., 2014) that compared Chuanxiong formulae with placebo, the data of migraine days in Chuanxiong formulae was significantly lower (MD = −0.74, 95% CI −1.30 to −0.18, *P* = 0.01, heterogeneity χ^2^ = 0.08, *P* = 0.78, *I*^2^ = 0%, Figure 4). For comparisons with CP, there was a benefit for the Chinese herbal medicine (CHM) group as well (MD = −0.50, 95% CI −0.80 to −0.20, *P* = 0.001, heterogeneity χ^2^ = 0.00, *P* = 1.00, *I*^2^ = 0%, Figure 4; She, 2013; Liang, 2015).

#### Pain intensity

**Figure 5:**
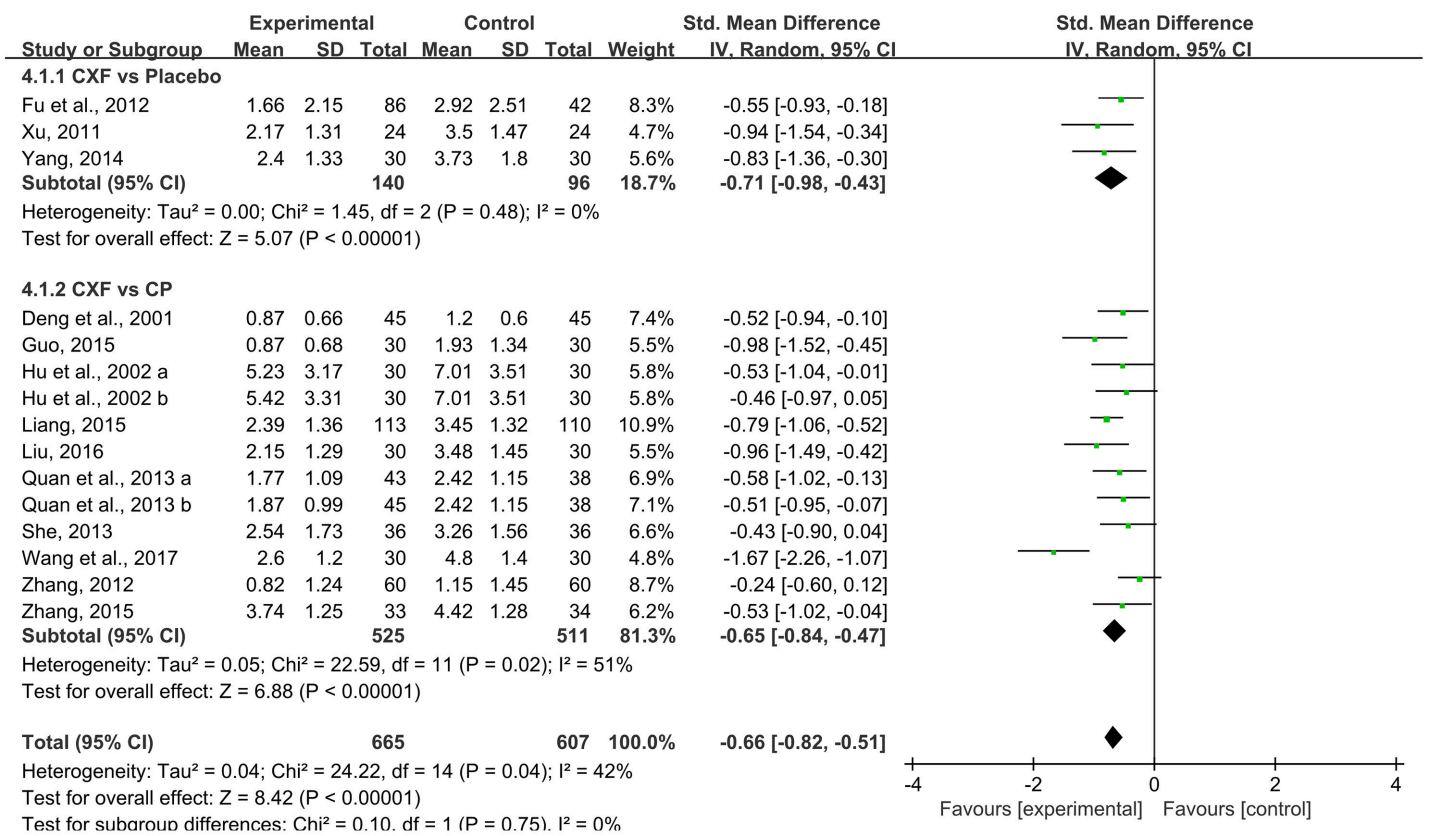
The forest plot of the efficacy of Chuanxiong formulae on pain intensity. CXF, Chuanxiong formulae; CP, conventional pharmacotherapy.

Pain intensity of migraine was observed in 14 studies. Pooled data showed that Chuanxiong formulae were significantly better at relieving the pain compared with placebo in 3 studies (SMD = −0.71, 95% CI −0.98 to −0.43, *P* < 0.00001, heterogeneity χ^2^ = 1.45, *P* = 0.48, *I*^2^ = 0%, Figure 5; Xu, 2011; Fu et al., 2012; Yang, 2014) and with CP in 10 studies (SMD = −0.67, 95% CI −0.84 to −0.47, *P* < 0.00001, heterogeneity χ^2^ = 22.59, *P* = 0.02, *I*^2^ = 51%, Figure 5; Deng et al., 2001; Hu et al., 2002; Zhang, 2012, 2015; Quan et al., 2013; She, 2013; Guo, 2015; Liang, 2015; Liu, 2016; Wang et al., 2017). One study (Zhang and Xu, 2017) indicated that the pain score of CHM plus CP groups was significantly lower than that of the CP group (*P* < 0.05).

#### The total clinical efficacy rate

**Figure 6:**
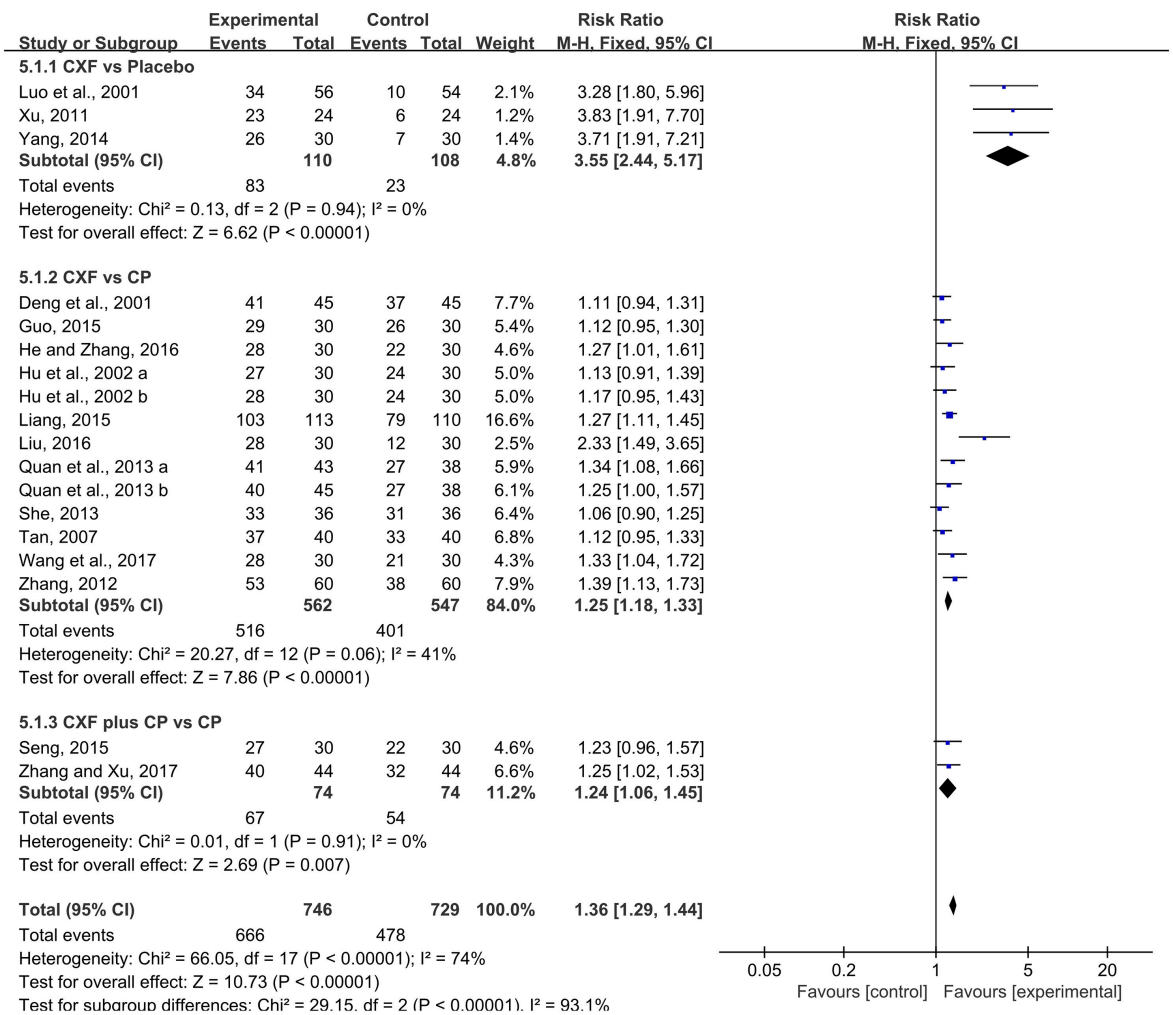
The forest plot of the efficacy of Chuanxiong formulae on the clinical efficacy rate. CXF, Chuanxiong formulae; CP, conventional pharmacotherapy.

The total clinical efficacy rate was reported in 16 studies with 18 comparisons. There were significant improvement comparing Chuanxiong formulae with placebo (RR = 3.55, 95% CI 2.44–5.17, *P* < 0.00001, heterogeneity χ^2^ = 0.13, *P* = 0.94, *I*^2^ = 0%, Figure 6; Luo et al., 2001; Xu, 2011; Yang, 2014). Compared with CP, the pooled data showed that Chuanxiong formulae was superior to CP (RR = 1.25, 95% CI 1.18–1.33, *P* < 0.00001, heterogeneity χ^2^ = 20.27, *P* = 0.06, *I*^2^ = 41%, Figure 6; Deng et al., 2001; Hu et al., 2002; Tan, 2007; Zhang, 2012; Quan et al., 2013; She, 2013; Guo, 2015; Liang, 2015; He and Zhang, 2016; Liu, 2016; Wang et al., 2017). Two studies (Seng, 2015; Zhang and Xu, 2017) showed that there was a benefit for the Chuanxiong formulae plus CP group when compared with CP (RR = 1.24, 95% CI 1.06–1.45, *P* = 0.007, heterogeneity χ^2^ = 0.01, *P* = 0.91, *I*^2^ = 0%, Figure 6).

### Adverse events

Sixteen out of 19 studies (Luo et al., 2001; Hu et al., 2002; Tan, 2007; Xu, 2011; Fu et al., 2012; Zhang, 2012, 2015; Quan et al., 2013; She, 2013; Cao et al., 2014; Yang, 2014; Guo, 2015; Seng, 2015; Liu, 2016; Wang et al., 2017; Zhang and Xu, 2017) reported the adverse events occurring during the treatment, in which a total of 61/742 (8.22%) patients suffered adverse events in the trial groups and 56/623 (8.99%) patients did so in control groups, and the rest three studies (Deng et al., 2001; Liang, 2015; He and Zhang, 2016) did not mention any information about adverse events. Ten studies (Tan, 2007; Xu, 2011; Zhang, 2012, 2015; Quan et al., 2013; Yang, 2014; Guo, 2015; Seng, 2015; Liu, 2016; Wang et al., 2017) stated that no adverse event happened during the treatment. In the 3 studies (Luo et al., 2001; She, 2013; Cao et al., 2014) with adequate information of adverse events, 40 cases reported that there were adverse reactions of the gastrointestinal reactions including indigestion, bloating and flatulence, epigastric pain, abdominal pain, constipation, vomiting and nausea in the experimental group, whereas it was occurred in 38 cases in the control group. Adverse reactions of nervous system such as somnolence, insomnia, dizziness is the second most frequent, 13 cases in trial groups and 15 cases in control groups. Adverse events of all studies were generally mild both in the Chuanxiong formulae and control groups. One study (Luo et al., 2001) reported that a patient suffered severe chest congestion and nausea, but the investigator did not consider the event to be related to study medication.

### Publication bias

**Figure 7:**
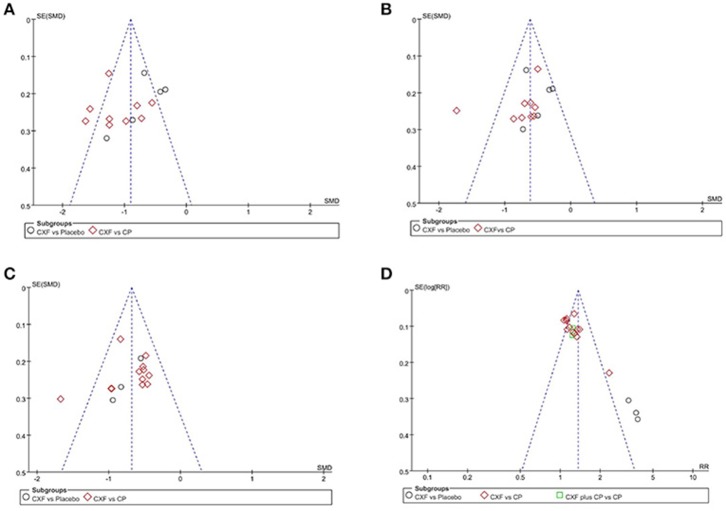
The funnel plots of the efficacy of Chuanxiong formulae on the migraine frequency **(A)**, migraine duration **(B)**, pain intensity **(C)**, and the clinical efficacy rate **(D)**. CXF, Chuanxiong formulae; CP, conventional pharmacotherapy.

Funnel plots were reviewed for four outcomes (Figure 7). The results showed symmetrical distribution for the outcomes of migraine frequency (Egger's test *t* = −1.17, 95% CI −6.58 to 1.95, *p* = 0.263), migraine duration (Egger's test *t* = −1.27, 95% CI −5.44 to 1. 42, *p* = 0.227), and pain intensity (Egger's test *t* = −0.96, 95% CI −4.79 to 1.82, *P* = 0.352), which did not suggest an obvious publication bias. However, there was a significant bias in the total clinical efficacy rate with Egger's test (*t* = 6.37, 95% CI 2.58 to 5.16, *p* < 0.001). Because the number of studies in the outcome of migraine days was limited (*n* = 4), funnel plot and Egger's test were not appropriate.

## Discussion

### Summary of evidence

A former review (Zhou et al., 2013) published in 2013 found some evidence of supporting the use of TCM for migraine; however the poor methodological quality and significant publication bias prevented the author making firm conclusions. Our previous review (Li et al., 2015) in 2015 also demonstrated that Chuanxiong Chadiao powder may be effective and safe for the treatment of headache. This is a systematic review of 19 high-quality RCTs with 1832 participants to determine the efficacy and safety of Chuanxiong formulae for migraine. The present study indicated that Chuanxiong formulae provided statistically significant benefits in terms of reducing frequency, duration, days, pain severity of migraine and improving the total clinical efficacy rate. In addition, Chuanxiong formulae appeared to be generally safe and well tolerated. Current evidence supported that Chuanxiong formulae could be an alternative drugs for the symptom treatment of migraine.

### Limitations

There are several limitations in the primary studies. Firstly, although we included the high-quality RCTs according to a cumulative score of at least 4 out of 7 for the Cochrane RoB tool domains, the methodological details was still not adequate in some studies. Only 3 studies (Xu, 2011; Fu et al., 2012; He and Zhang, 2016) described a proper method of allocation concealment and 5 studies (Luo et al., 2001; Xu, 2011; Fu et al., 2012; Cao et al., 2014; Yang, 2014) employed the blinding procedure. Some studies were unable to be blinded, due to the fact that TCM is special in color, smell and taste, in contrast to the standard capsule of Flunarizine Hydrochloride. However, no study used a double-dummy technique to reduce the difference of drugs between the experiment and control groups. Blinding makes it difficult to bias results intentionally or unintentionally and helps ensure the credibility of study conclusions (Day and Altman, 2000). In addition, the intervention of trials with inadequate allocation concealment is 18% more “beneficial” than in trials with adequate concealment (Higgins and Green, 2011). Secondly, migraine affects approximately 18% of women and 6% of men (Lipton et al., 2007). The ratio of gender is amplified in the included RCTs. This gender selection bias should be avoided by recruiting males to an extent. Thirdly, relatively long treatment periods could increase the power of the trial by providing more stable estimates for the efficacy of Chuanxiong formulae. However, the treatment duration ranged from 1 to 16 weeks. The long-term safety of Chuanxiong formulae for headache could not be determined because follow-up period in the studies ranged from 1 week to 6 months. Guidelines for controlled trials of drugs in migraine recommends that treatment periods is no less than 3 months in phase II RCTs and up to 6 months in phase III trials, and every 4 weeks visits is necessary (Tfelt-Hansen et al., 2012). Fourthly, due to the context in terms of traditional culture and the barrier of language, all RCTs were in English or in Chinese and have been conducted in Chinese population, which restricts the generalizability of the findings. Fifthly, migraine treatment can be divided into acute treatment and preventive treatment (Antonaci et al., 2016). It is difficult to differentiate the effectiveness of Chuanxiong formulae in two kinds of treatments because the weakness rooted in primary studies. In fact, acute treatment is focused on single episodes of headache and no RCTs were designed specifically for acute treatment of Chuanxiong. Thus, further particular trial design of acute treatment of Chuanxiong is needed.

### Implications for practice

The use of TCM in treating many common neurological ailments has been paid more attention over the years (Ma et al., 2009). Chuanxiong is widely used in TCM for headache. The main active ingredients of Chuanxiong for migrain include tetramethylpyrazine (TMP), senkyunolide A, ferulic acid (FA) and ligustilide (Ran et al., 2011). The significant pharmacological activities of Chuanxiong and its main compounds are as follows: (1) Antioxidant effects: TMP, FA and ligustilide could reduce the production of intracellular reactive oxygen species (ROS) and nitric oxide (NO), and the expression of inducible nitric oxide synthase (iNOS) (Wong et al., 2007; Chung et al., 2012; Zheng Z. et al., 2013; Cao et al., 2015; Ren et al., 2017). TMP and FA inhibit the activity of NADPH oxidase via ERK signaling pathway and NF-κB pathway respectively (Wong et al., 2007; Cao et al., 2015). (2) Antiinflammatory effects: TMP, senkyunolide A and ligustilide could down regulate the activation and proliferation of astrocytic, the production and bioactivity of tumor necrosis factor α (TNF-α), and the expression of cyclooxygenase-2 (COX-2) protein (Liu et al., 2005; Chung et al., 2012; Feng et al., 2012; Jiang et al., 2017). (3) Antiapoptotic effects: Ligustilide prevented neuronal apoptosis in both parietal cortex and hippocampus through regulation of mitochondrion metabolism (Feng et al., 2012) TMP could decrease the levels of miR-214-3p and increase the expression level of Bcl2l2 (Fan and Wu, 2017). FA was mainly through TLR4/MyD88 signaling pathway and NF-kB pathway (Cao et al., 2015; Ren et al., 2017). (4) Antinociceptive effects: TMP could inhibit the expression of P2X3 receptor in the trigeminal ganglia (TG), exhibiting potential effect on pain relief (Xiong et al., 2017). Ligustilide could activate the transient receptor potential cationic channel ankyrin 1 (TRPA1) (Zhong et al., 2011) and display high affinities with 5-hydroxytryptamine (5-HT) 1D receptors (Du et al., 2015) and 5-HT 7 receptors (Deng et al., 2006), regulating the release of calcitonin gene-related protein (CGRP) which can cause vasodilatation. Thus, Chuanxiong formulae are likely to be multi-targeting therapy for the multi-hit driven migraine pathogenesis. However, it remains to clarify the nature of the ingredients of the mixture and the mechanisms of action of Chuanxiong. This should be the object of further studies.

### Implications for further studies

Firstly, we suggested that the protocol of clinical trials must register in clinical trials registry platform and CONSORT 2010 statement should be applied in trial reporting and publication. Secondly, in order to facilitate more reliable comparison of study results, more consistency in the use of the international standard on migraine clinical trials, such as guidelines for controlled trials of drugs in migraine: 3rd edition, which consist of the following parts: selection of patients, trial design, evaluation of results and statistics (Tfelt-Hansen et al., 2012). The type of migraine should be illustrated definitely in trials, which could give precise evidence for clinic. Meanwhile, we also recommend the appropriate sample size that calculated before enrollment, ideal length of treatment and follow-up, adequate randomization methods, sufficient blinding, and intent-to-treat (ITT) analyses in future RCTs. Thirdly, Radix Angelicae Dahuricae, Ramulus Uncariae Cum Uncis, Herba Asari, Radix Angelicae Sinensis, and Scorpio were the most frequently used herbs, which should be considered firstly when formulating optimal combination of Chuanxiong with other herbal ingredients. Finally, the exact pathomechanism of migraine and the pharmacological mechanism of Chuanxiong remain largely unknown, which should be further investigated.

## Conclusion

The present findings indicated that Chuanxiong formulae provided statistically significant benefits for migraine and were generally safe. Thus, the available evidence of present study supported the alternative use of Chuanxiong formulae for migraine.

## Author contributions

Study conception and design: GZ and CS; Acquisition, analysis and/or interpretation of data: CS, QX, YS, YW, ZH and GZ; Final approval and overall responsibility for this published work: GZ.

### Conflict of interest statement

The authors declare that the research was conducted in the absence of any commercial or financial relationships that could be construed as a potential conflict of interest. The reviewer EP and handling Editor declared their shared affiliation.

## Abbreviations

5-HT: 5-hydroxytryptamine
CAM: complementary and alternative medicine
CGRP: calcitonin gene-related protein
CHM: Chinese herbal medicine
CI: confidence intervals
CNKI: China National Knowledge Infrastructure
COX-2: cyclooxygenase-2
CP: conventional pharmacotherapy
FA: ferulic acid
FEM: fixed effect model
GBD: global burden of disease.
GDP: gross domestic product
ICHD-1: Classification and Diagnostic criteria for headache disorders, cranial neuralgias and facial pain
ICHD-2: The international classification of headache disorder, 2nd edition
ICHD-3: The international classification of headache disorder, 3rd edition
ID: identity
iNOS: reactive oxygen species
ITT: intent-to-treat
MD: mean difference
miR-214-3p: microRNA-214-3p
MOH: medication-overuse headache
NO: nitric oxide
NSAIDs: non-steroidal anti-inflammatory drugs
RCTs: randomized controlled trials
REM: random effect model
RoB: risk of bias
ROS: reactive oxygen species
RR: relative risk
SAS: Statistical Analysis System
SMD: standardized mean difference
SPSS: Statistical Product and Service Solutions
TCM: traditional Chinese medicine
TG: trigeminal ganglia
TMP: tetramethylpyrazine
TNF-α: tumor necrosis factor α
TRPA1: transient receptor potential cationic channel ankyrin 1.
VIP: Chinese Science and Technology Periodical Database.
